# Recent Progress in Anæsthetics

**Published:** 1910-05-28

**Authors:** 


### SPECIAL ARTICLE.

### RECENT PROGRESS IN ANÆSTHETICS.

The most important event this year in the anæsthetic world, or at least in the British section of it, has been the publication of the report of the Home Office Committee appointed to inquire into the question of deaths resulting from the administration of anæsthetics, issued on April 9. The conclusions adopted are numerous and important, for the Committee sum up their recommendations thus:—

Every death under an anæsthetic should be reported to the coroner, who, after inquiry, should determine whether it is desirable to hold an inquest or not. In the case of every death under an anæsthetic the medical certificate of death should specify the fact whether the anæsthetic was the actual cause of death or not.

No general respirable anæsthetic should be administered by any person who is not a registered medical or dental practitioner. Registered dentists should be confined to the use of nitrous oxide gas, and should not employ the general respirable anæsthetics.

Intra-spinal anæsthesia should be practised only by registered medical practitioners.

Practical and theoretical instruction in the administration of anæsthetics should be an essential part of the medical curriculum. Such instruction in the administration of gas should be an essential part of the dental curriculum.

In the case of any death under an anæsthetic in a hospital or other similar public institution there should be a scientific investigation into the actual cause of death conducted by the authorities of the institution. A small standing scientific committee on anæsthetics should be instituted under the authority of the Home Office.

The Committee state that deaths under anæsthetics have risen from five in 1866 to 155 in 1905. While no absolute deductions can be drawn from these figures in that deaths under anæsthetics may come from a variety of causes, and since moreover the number of operations performed to-day shows an enormous increase over that of forty years ago, still it is a fact that there is an increasing number of deaths under anæsthetics, and in the opinion of experts a certain number of these deaths are due to preventable causes. The Committee lay stress on the fact that as the law stands anæsthetics may be administered by persons who possess no qualifications of any kind, and they feel very strongly that this unregulated state of affairs constitutes a serious menace to the public. With regard to the administration of anæsthetics by dentists, it is urged that in the use of nitrous oxide gas in no case should one person both administer the gas and perform the operation. If a medical man be not employed, a registered dental practitioner should be called in for this purpose.

With these recommendations taken as a whole the medical profession can have very little disagreement. They do not go quite so far as some of those who have been ventilating the matter would like, and the limitation of dental surgeons to nitrous oxide gas alone among general anæsthetics may displease some of the more advanced practitioners of that branch of surgery. Since, however, dentists are every year using local methods of anæsthesia more, and general respirable anæsthetics less, opposition is much less likely to be serious than it would have been a few years ago. But taking them all round, it must be admitted that the protection as well as the convenience of the public have been throughout kept first in view, and it is at least matter for congratulation that the present extremely unsatisfactory state of the law is attracting attention among the public as well as the profession. It remains for the Home Office to translate these conclusions into law. Unfortunately the constitutional turmoil now proceeding distracts not only public opinion from questions of this kind, but also induces Ministers to go electioneering instead of sticking to their offices. Past experience proves that only by renewed and continual pressure can Governments be induced to adopt even the most obvious and necessary measure of reform which has no vote-producing value. The profession must by united effort combine to activate the Home Office until the Committee's conclusions are embodied in legislative form.

### Blood-pressure.

Several investigators have been busy over the conditions of the circulatory system altered or set up by different anæsthetics. Drs. Guy, Goodall, and Reid describe their researches [1] into nitrous oxide gas and ethyl chloride from this aspect. They find that there is no practical difference between the available anæsthesia brought about by nitrous oxide gas when rebreathing is allowed and that available when given with valves, but the effects of asphyxia are much more marked in the latter method. The asphyxial element in the rebreathing method can be avoided by the previous breathing of a gallon of oxygen, but the period of available anæsthesia is then often shortened by a few seconds.

Ethyl chloride causes marked disturbance of the circulation, and in large doses may bring about a dangerous fall of systolic blood-pressure by inhibition of the heart. This fall is prevented by the previous inhalation of gas; the effect then is a constant rise, probably in part due to asphyxia. The slight asphyxial element in the gas-ethyl-chloride sequence can be eliminated by the previous administration of a gallon of oxygen, and this does not shorten the period of available anæsthesia.

### American Papers.

In a paper relating to the prevention of surgical shock by attention to details of operative technique, Dr. Rakestraw lays stress [2] on the value of fresh air. He has his patients placed on a verandah in the open air while they are coming round from the anæsthetic; this, he thinks, often relieves nausea immediately and enables the patient to recover from the anæsthetic in a very short time. The re-inhalation of ether in a closed room and the constant smelling of its odour prolongs nausea, and if the patient is well protected from dust, noises, and interference generally, the idea seems to be distinctly a good one. Sunlight he also believes in as an agent which has a similar soothing effect.

From the Sloane Maternity Hospital comes a strong protest against the use of chloroform for the control of eclamptic convulsions. It has struck Dr. Ward and others of the staff there that since there is a marked similarity between the symptoms of delayed chloroform poisoning and of the toxæmias of pregnancy, the use of chloroform is contra-indicated whenever there are signs of a toxæmia, however slight. He believes that by this method the number of patients who have had eclampsia—that is, who have actually been convulsed—has been much reduced. One authority quoted, Dr. Ewing, even thinks that a considerable proportion of cases of toxæmia of pregnancy are really cases of delayed chloroform poisoning. Ether is advised for the actual delivery or for necessary manipulations whenever an anæsthetic is required; but to give an anæsthetic for the sole purpose of modifying the fits is described as simply controlling a symptom without affecting the cause of the disease.

According to Dr. Ward, the patients take ether well, and there is no difficulty in its use. It has been objected that when the kidneys are much diseased ether may irritate them more than chloroform, and so do harm. But the author has not found it so in practice, and believes that a larger share of the trouble is always to be traced to the liver than to the kidneys. Others have thought that ether may affect the liver just as chloroform sometimes does, but this view also remains without clinical confirmation. Apart from the control of convulsions, it may be well to mention that all the usual accepted treatment of eclampsia is adopted: emptying of the uterus as soon as possible without risk of lacerating the cervix and vagina; hot packs and baths; colon irrigation; nitroglycerin, veratrum viride, and chloral.

[1] *Edinburgh Medical Journal*, March 1910.
[2] *American Journal of Obstetrics and Gynœcology*, March 1910, p. 407.

